# Characterization of UDP-glucuronosyltransferase (UGT1A1) Promoter Polymorphisms and Gene Expression on Ethnicity, Stage of Disease, and Menopausal Status in Breast Cancer

**DOI:** 10.4172/2157-7609.S4-001

**Published:** 2012-03-19

**Authors:** Athena Starlard-Davenport, Beverly R. Word, Beverly Lyn-Cook

**Affiliations:** Office of the Associate Director for Regulatory Activities, National Center for Toxicological Research, Jefferson, AR 72079, USA

**Keywords:** UGTs, UGT1A1, Breast cancer, Genetic polymorphism

## Abstract

Estrogen metabolism, catalyzed by UGTs, is a major drug-metabolic pathway that results in inactivation of estrogens and their metabolites. Alterations in UGTs involved in estrogen metabolism, has been suggested to play a role in breast cancer risk. The purpose of this study was to: 1) compare the mRNA expression levels of UGTs involved in estrogen metabolism in human breast tissues from women; 2) compare UGT1A1 mRNA expression to tumor stage, ethnicity, and menopausal status in a group of human breast tumors and normal breast tissues, and 3) investigate the association between variations in the number of TA repeats in the promoter region of UGT1A1 to gene expression. Quantification of UGT mRNA in breast tissues revealed that UGT1A4, UGT1A10, and UGT2B7 mRNA levels were decreased in breast cancers as compared to normal breast tissues. UGT1A1 mRNA levels were also significantly decreased in breast cancers as compared to normal breast tissues (Tumor: 0.5 ± 0.2; Normal: 4.1 ± 1.3, *p* = 0.0006). UGT1A1 mRNA down-regulation was strongly correlated with postmenopausal status in breast cancer versus controls (*p* = 0.04). In all the UGT1A1 genotypes observed in our study, the mean mRNA levels was significantly decreased among breast cancer cases as compared to controls for UGT1A1*1/*1 (*p* = 0.004), UGT1A1*28/*28 (*p* = 0.03) and UGT1A1*28/*37 (*p* = 0.06). Our findings demonstrate that further investigations are necessary to determine the role of UGT1A1 in breast carcinogenesis.

## Introduction

Breast cancer is the most common malignancy in women in the United States [1]. Despite recent advancements in breast cancer research, including breast cancer staging as a prognostic marker, it is still difficult to predict optimal therapy, breast cancer recurrence, and clinical outcome [2–4]. A major risk factor for breast cancer development in women is association with exposure to increased concentrations of estrogens over a prolonged period of time [5]. The standard paradigm to account for this association focuses on increased cell proliferation caused by estrogen through estrogen receptor (ER)-mediated signal transduction accompanied and cellular oxidative metabolism of estrogens [6–8].

One of the major pathways involved in estrogen inactivation and removal from circulation is through the process of glucuronidation, catalyzed by UDP-Glucuronosyltransferases (UGTs) [9]. UGTs have the catalytic capacity to inactivate estrogens and their oxidative metabolites to inactive metabolites [10–12]. UGTs also maintain the steady state concentrations of estrogens, and other sex steroids, in breast tissues [13]. Thus, suggesting that UGTs play a protective role in the breast by terminating or attenuating the biological activities of estrogens and its metabolites. However, modifications in the glucuronidation metabolic pathway, through loss of UGT expression and activity, have been proposed to alter estrogen metabolism, resulting in an increased accumulation of genotoxic estrogen metabolites and risk of breast cancer development [14–16]. For instance, a genetic variant in UGT1A1, which is characterized by the number of TA repeats in the TATA box of the promoter region, has been associated with reduced transcriptional activity towards estrogens *in vitro* and increased breast cancer risk in several population-based studies [17–20]. The UGT1A1 variant allele that is associated with reduced transcriptional activity is A(TA)_7_TAA and A(TA)_8_TAA (*28 and *37) and the wildtype allele that is associated with enhanced transcriptional activity is A(TA)_5_TAA and A(TA)_6_TAA (*36 and *1, respectively). The frequency of TA repeats has been reported to also vary among different ethnic groups [20] and ER status [18].

The present study was, thus, undertaken to: 1) investigate the mRNA expression of UGTs involved in estrogen metabolism; 2) compare UGT1A1 mRNA expression to potential clinical relevance (i.e. associations with tumor stage), ethnicity, and menopausal status in a group of human breast tumors and non-tumor (normal) breast tissues, and 3) investigate the association between variations in the number of TA repeats in the promoter region of UGT1A1 with gene expression.

## Materials and Methods

### Human breast tissues

Human breast tissues from women were purchased from the Cooperative Human Tissue Network (Birmingham, AL, USA). Tissue samples (*n* = 92) were snap-frozen and stored at −80ºC until analysis. A total of 57 breast cancers (cases) and 35 normal (controls) breast tissues from women undergoing mammoplasty for macromastia or fibrocystic tissue were genotyped in the present study. Characteristics of donors used in these studies are detailed in Table 1. Authorization for the use of these tissues for research was obtained from the RIHSC at the National Center for Toxicological Research, Jefferson, AR.

### Genotyping analysis

Genotyping was performed on genomic DNA extracted from human tissue samples using the Qiagen DNA Isolation Kit (Valencia, CA), following the manufacturer’s protocol. Polymerase chain reactions (PCR) were conducted in a total volume of 50 μL containing 100 ng of genomic DNA, 25 pmoles of the UGT1A1 primer (UGT1A1 forward: 5′-GAT TTG AGT ATG AAA TTC CAG CCA G-3′ and UGT1A1 reverse: 5′-CCA GTG GCT GCC ATC CAC T-3′) [21] and the following reagents (obtained from Promega, Madison, WI): 0.1 mM each of dCTP, dGTP, dATP and dTTP; 2.5 mM of MgCl_2_; 50 mM of potassium chloride; 10 mM of Tris (pH 9.0); 0.1 % Triton-X; and 2.5 U of Taq polymerase. After 35 cycles of amplification (denaturation at 94°C for 30 sec, annealing at 60°C for 1 min, and extension at 72°C for 1 min), the amplification products were electrophoresed in 3% agarose gel and visualized after staining with ethidium bromide.

Genotypes of the A(TA)_6–8_TAA polymorphism were determined by bidirectional sequencing with forward or reverse primers. PCR products were purified using the Qiagen DNA Purification Kit (Valencia, CA). DNA of the UGT1A1 product (351 bp) containing the polymorphic TA repeats was performed with a dye terminator sequence reaction (ABI Prism DNA Sequencing Kit; Perkin-Elmer, Foster City, CA) using an ABI PRISM 310 DNA Sequencer. UGT1A1 genotypes were assigned based on the number of TA repeats for each allele (i.e., 6/6, 6/7, 7/7, or 7/8).

### RNA isolation and quantitative reverse transcription realtime PCR (qRT-PCR)

Total RNA was extracted from human breast tissues using the RNeasy Mini Kit (Qiagen, Valencia) according to the manufacturer’s instructions. RNA isolated from breast specimens were dissolved in RNAse-free water and quantified by spectrophotometric readings at 260 nm (*A*_260_). The quality of total RNA was determined by the *A*_260_/ *A*_280_ ratio and their integrity was confirmed using Experion System (Bio-Rad, Hercules, CA). Reverse transcription was performed using a High Capacity cDNA Reverse Transcription Kit (Applied Biosystems, Foster City, CA). The levels of UGT1A1, UGT1A4, UGT1A7, UGT1A8, UGT1A10, and UGT2B7 gene transcripts were determined utilizing TaqMan Gene Expression Assays (Applied Biosystems, Foster City, CA) according to the manufacturer’s instructions. Relative transcript levels were determined using Human *B*-actin Endogenous Control (Applied Biosystems, Foster City, CA) as an internal reference. Differences between the breast tissue samples were determined using comparative delta C_T_ method [22]. All qRT-PCR reactions were performed blind to sample information, in triplicate, and repeated twice.

### Statistical analysis

All statistical analyses were performed at the completion of this study using GraphPad Prism, version 4 software (La Jolla, CA). Data were expressed as the mean ± standard deviation. Relative UGT mRNA expression in tumor and in normal breast tissue in each group was transformed using a common logarithm. For statistical comparisons of log-transformed data between two groups, a nonparametric *t*-test was used accordingly. The Mann–Whitney U-test and Kruskal–Wallis test was used to correlate mRNA expression of UGT1A1 with ethnicity, menopausal status, and tumor stage. For all of the statistical tests, a twosided *p*-value of less than 0.05 was considered statistically significant. Normal and cancer specimens for which UGT mRNA expression was not detected by quantitative real-time PCR were included for statistically analysis.

## Results

### mRNA expression of UGT isoforms

**Figure 1:**
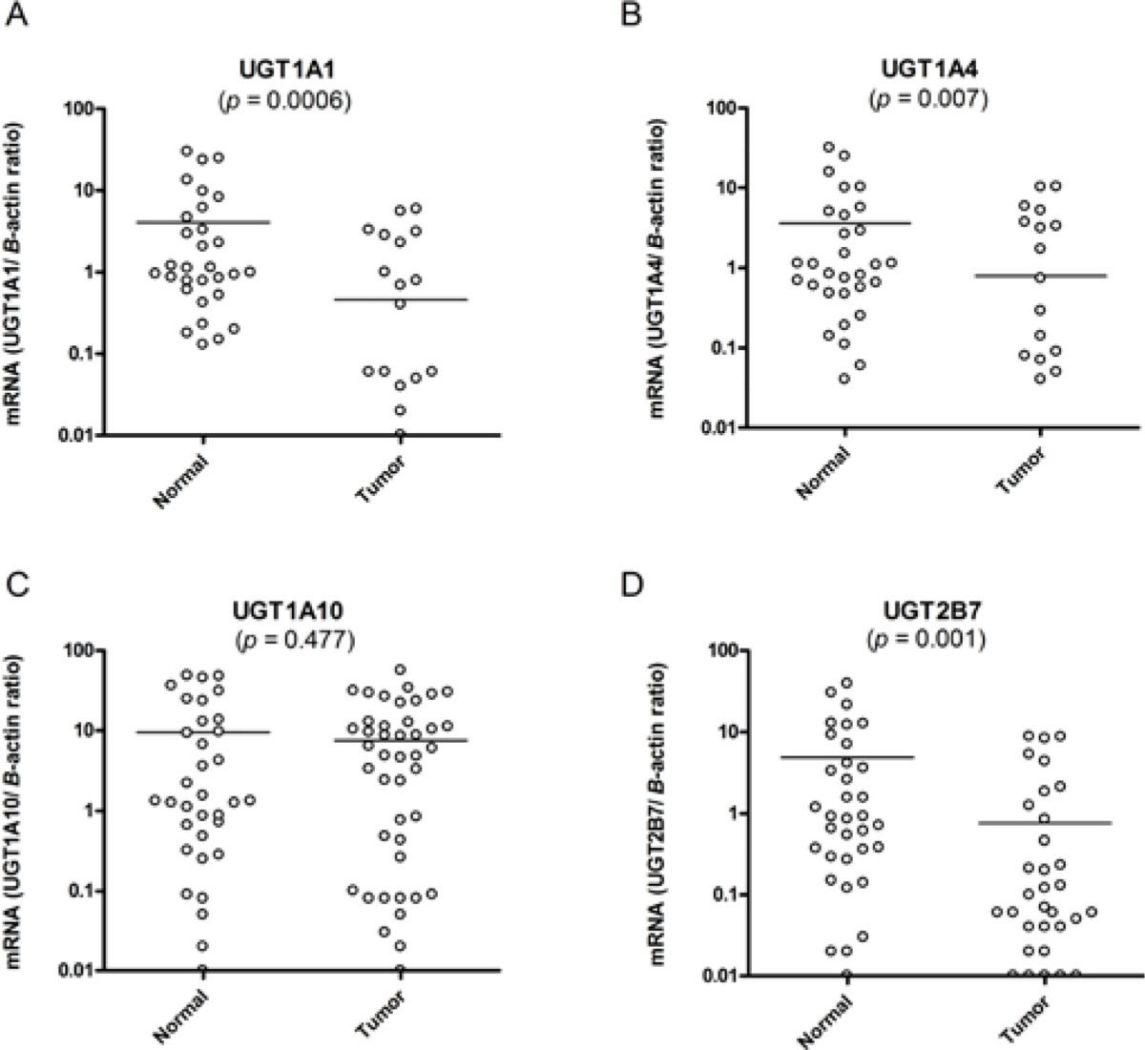
mRNA expression of UGTs in human breast samples relative to *B*-actin. Quantitative real-time PCR analysis of UGT1A1, UGT1A4, UGT1A10, and UGT2B7 mRNA expression in normal and tumor breast tissues. Solid horizontal lines demonstrate the mean with the 95% CI of each individual group. All data are shown as the ratio between the UGT target gene and *B*-actin internal control on a logarithmic scale. *p* -value, nonparametric, Mann-Whitney tests. *p<0.05, **p <0.01 is statistically significant.

Quantitative real-time PCR was used to analyze the mRNA expression of UGT1A1, UGT1A4, UGT1A8, UGT1A10, and UGT2B7 in 57 breast tumor specimens and 35 normal (control) specimens with no evidence of malignancy (Figure 1). The CT values for *B*-actin mRNA expression among all donors did not vary significantly (data not shown). The mRNA expression of all UGTs studied, with the exception of UGT1A8, was detected in the majority of normal breast tissue specimens; however, several UGT mRNAs were either down-regulated or not expressed in the majority of breast cancers. Specifically, UGT1A1 and UGT1A4 were not expressed in 5 normal breast specimens and 40 and 39 breast cancers, respectively. UGT1A10 and UGT2B7 were not expressed in 1 normal breast specimen and 12 and 25 breast cancers, respectively. Wide individual variations in transcript levels among the UGTs were analyzed. Furthermore, the mean mRNA expression levels of UGT1A4 (Tumor: 0.8 ± 0.3; Normal: 3.6 ± 1.2, *p* = 0.007), UGT1A10 (Tumor: 7.5 ± 1.3; Normal: 8.8 ± 2.4, *p* = 0.6), and UGT2B7 (Tumor: 0.8 ± 0.3; Normal: 4.9 ± 1.5, *p* = 0.001) was significantly down-regulated in breast tumors as compared to normal breast tissues. Of the UGTs analyzed, the mean UGT1A1 mRNA expression levels were significantly lower in breast cancer specimens as compared to normal breast tissues (Tumor: 0.5 ± 0.2; Normal: 4.1 ± 1.3, *p* = 0.0006). UGT1A4 (*p* = 0.007) and UGT2B7 mRNA expression (*p =* 0.001) both showed a similar decrease in mRNA expression pattern as UGT1A1 in breast tumor specimens; however, the mean mRNA levels of UGT1A1 was most significantly decreased in breast cancers compared to normal controls (*p* = 0.0006) compared to UGT1A4 (*p* = 0.0068) and UGT2B7 (*p* = 0.0014). Based upon these findings, we conducted further analysis to determine if UGT1A1 mRNA levels have an effect on clinicopathological factors, stage, ethnicity and menopausal status.

### Comparison of UGT1A1 mRNA expression to ethnicity, menopausal status and stage of breast disease

**Figure 2:**
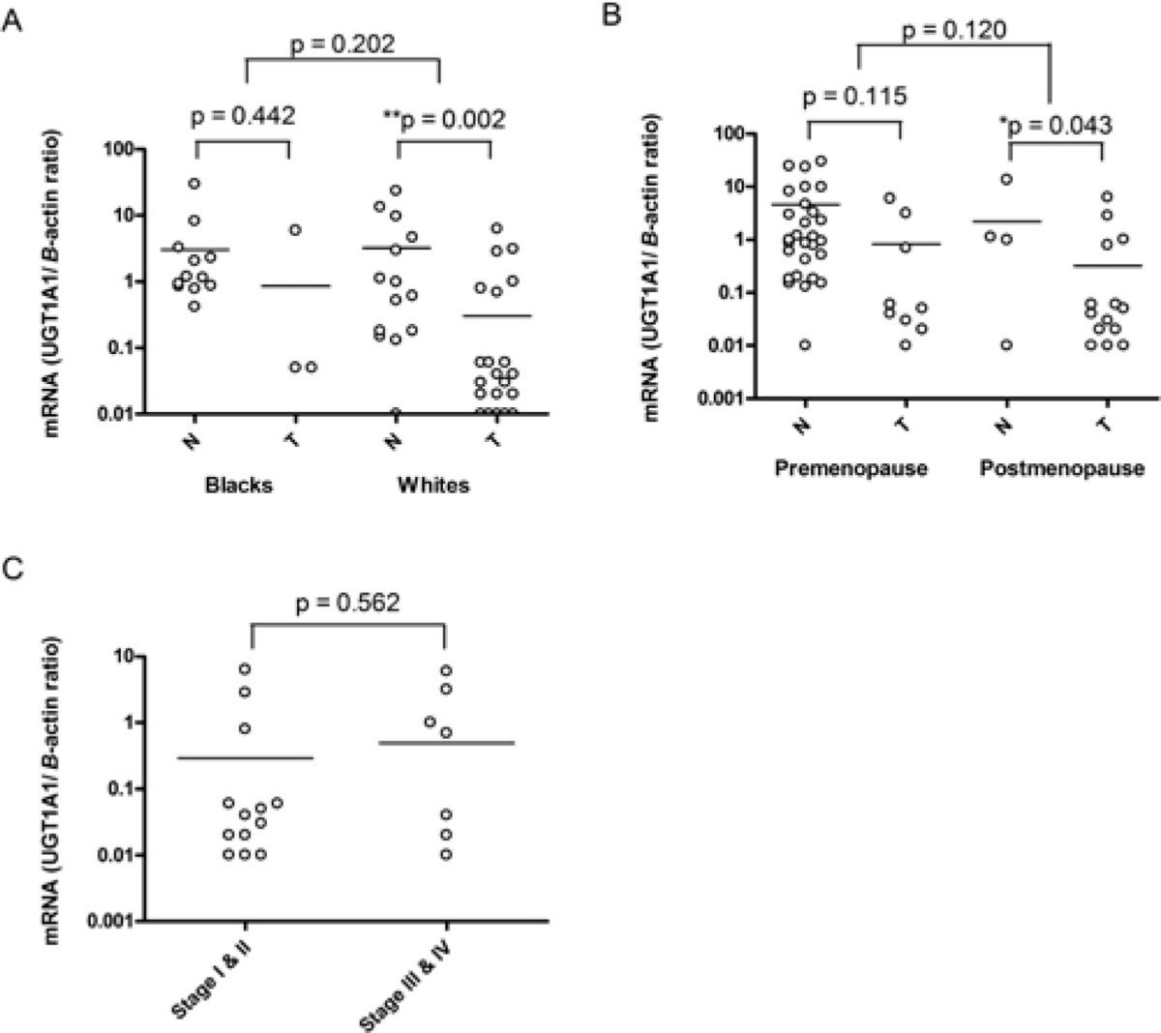
Effect of UGT1A1 mRNA expression in human breast samples on ethnicity, menopausal status, and stage of breast disease. Quantitative real-time PCR analysis of UGT1A1 mRNA expression in normal (N) and tumor (T) human breast tissues and its effect on ethnicity (A), menopausal status (B), and stage of breast disease (C). Solid horizontal lines demonstrate the mean with the 95% CI of each individual group. All data are shown as the ratio between the UGT target gene and *B*-actin internal control on a logarithmic scale. *p* -value, nonparametric, Mann-Whitney or Kruskall-Wallis tests. *p < 0.05 is statistically significant.

A multivariate analysis was done to determine factors with which UGT1A1 mRNA expression was associated. The effect of UGT1A1 mRNA levels on ethnicity, menopausal status, stage of breast disease, and ER status are shown in Figure 2. Although the mean UGT1A1 mRNA expression was not significantly different among ethnic groups, UGT1A1 mRNA expression levels was lower in breast cancer cases vs controls among EAs (Tumor: 0.3 ± 0.1; Normal: 3.2 ± 1.5, *p* = 0.002) (Figure 2A). Although not significant, UGT1A1 mRNA expression levels were still lower in breast cancer cases versus controls among AAs, (Tumor: 0.9 ± 0.8; Normal: 3.0 ± 1.7, *p* = 0.44) (Figure 2A). However, there was a significant difference in UGT1A1 mRNA in postmenopausal women breast cancer cases compared to controls (Tumor: 0.3 ± 0.2; Normal: 2.2 ± 1.9, *p* = 0.04) (Figure 2B). There was no significant difference overall in UGT1A1 mRNA levels between premenopausal and postmenopausal breast cancer cases versus controls (*p* = 0.12) (Figure 2B).

We also compared the expression levels of UGT1A1 mRNA to tumor stage. Interestingly, UGT1A1 mRNA levels were slightly higher, but not significantly, in more aggressive and advanced stages of breast disease as compared to early stages of breast disease (Stage I/II: 0.3 ± 0.2; Stage III/IV: 0.5 ± 0.3, *p* = 0.56) (Figure 2C).

### Distribution of UGT1A1 genotypes and comparison to UGT1A1 gene expression

Results of the genotype frequencies of the UGT1A1 promoter polymorphism among breast cancer cases and controls are presented in Table 2. A total of 57 breast cancer cases and 35 controls were genotyped for the UGT1A1 TATA box promoter polymorphism. In our study, we did not observe the UGT1A1*36/*1 genotype, which is more prevalent among AA [18]. We observed that the UGT1A1*1/*1 was most prevalent among breast cancer cases (58%) and controls (57%), followed by UGT1A1*28/*28 (Cases: 23%, Controls: 17%).

We postulated that the decreased levels and lack of UGT1A1 mRNA in breast cancers might be due to a genetic variant in the promoter region of UGT1A1. Therefore, we investigated the effect of the UGT1A1 (TA) repeat promoter polymorphism on UGT1A1 mRNA expression among breast cancer cases compared to controls. The means comparing UGT1A1 gene expression to UGT1A1 genotypes for breast cancer cases and controls are presented in Table 3. In breast cancer cases with the UGT1A1*28/*37 genotype, which represents low activity alleles, the mean UGT1A1 mRNA levels was lowest (*0.01 ± 0.02*). Furthermore, among all UGT1A1 genotypes identified, the mean UGT1A1 transcript levels were significantly decreased among breast cancer cases compared to controls (p=0.04). In all UGT1A1 genotypes observed in our study, the mean UGT1A1 mRNA expression levels was significantly decreased among breast cancer cases as compared to controls (p=0.04).

## Discussion

Excessive exposure to estrogen over a prolonged period of time is a causal factor of breast carcinogenesis [5]. Estrogen metabolism by UGTs is the major drug-metabolic pathway that results in the complete inactivation of estrogens and their hydroxylated metabolites [9–12]. Alterations in UGTs, resulting in decreased UGT expression and glucuronidation activity towards estrogens and their metabolites, has been suggested to play a potential role in breast cancer risk [14–16]. Our previous published study demonstrated that several UGTs, including UGT1A1, are involved in the complete inactivation of both native estrogens and their oxidative metabolites, including the genotoxic 4-OH-E1 [12]. We also demonstrated for the first time that UGT1A10 was the major UGT involved in estrogen and estrogen metabolite conjugation [16]. Furthermore, we showed that several UGTs, namely UGT1A10 and UGT2B7 were differentially expressed in non-malignant and malignant human breast tissues from AA and EA women [16]. In the present study, we characterized the mRNA expression of UGT1A1, UGT1A4, UGT1A8, UGT1A10, and UGT2B7 in normal and breast tumor tissues from AA and EA women. Our results demonstrate that the mRNA levels of the UGTs analyzed, with the exception of UGT1A8, which was not expressed, are differentially down-regulated in breast cancers as compared to normal breast tissue specimen from AA and EA women in this study. This finding is in agreement with our published study in 2008 [16]. We also showed that UGT1A1 mRNA is significantly decreased in breast tumors compared to normal breast tissues. UGT2B7 and UGT1A4 showed a similar down-regulated pattern in breast cancers compared to normal breast tissues. In support of our findings, an epidemiological study, demonstrated that the presence of lower levels of UGT1A1 protein resulted in decreased production of estrogen glucuronides and consequently higher exposure to E_2_ [17]. Furthermore, a study by Gestl et al. demonstrated that UGT2B7 mRNA and protein was downregulated in breast tumors as compared to normal breast tissues [14], which is consistent with our results in this study. To further determine if UGT1A1 mRNA levels had an effect on clinicopathological and variable factors, we compared UGT1A1 mRNA expression levels to ethnicity, menopausal status, and stage of breast disease. Although the number of specimens analyzed limited our study, we still observed a significant decrease in UGT1A1 mRNA levels among postmenopausal breast cancer cases versus controls and among EA women with breast cancer compared to EA without breast cancer. In support of our findings, a large population study demonstrated that the UGT1A1 (TA) repeat promoter polymorphism was significantly associated with increased breast cancer risk in postmenopausal women [23]. We also demonstrated that UGT1A1 mRNA was slightly increased in Stage III and IV breast cancers. This increase in UGT1A1 mRNA levels in more aggressive stage of breast disease could be attributed to epigenetic regulation of UGT1A1 resulting in variations in response to chemotherapy used for the treatment of more aggressive cancers [24]. The finding of a significant decrease of UGT1A1 mRNA levels in breast tumor specimens from postmenopausal women suggests that UGT1A1 may play a vital role in estrogen conjugation. The interindividual differences observed for UGT1A1 mRNA expression in breast cancer tissues compared to normal breast tissues with respect to ethnicity, menopausal status, and stage of breast disease further suggest that genetic variations or promoter polymorphisms in UGT1A1 may be involved. Our study demonstrated that UGT1A1 gene expression is decreased in breast cancer cases in comparison to controls in AA and EA female donors who have either the UGT1A1*1/*1, UGT1A1*1/*28, UGT1A1*28/*28, or the UGT1A1*28/*37 genotype. Several populationbased studies have observed differences in the TATA box promoter polymorphisms of UGT1A1 in regards to ethnicity, menopausal, and ER status [15,18,23]. In the study by Guillemette et al. [18] which analyzed 200 AA women with invasive breast cancer and 200 matched controls for the UGT1A1 TA repeat promoter polymorphism, they demonstrated that among pre-menopausal AA women, the association was stronger for estrogen receptor (ER)-negative breast cancers (OR 2.1, 95% CI 1.0–4.2; *P* = 0.04) than for ER-positive breast cancers (OR 1.3, 95% CI 0.6–3.0; *P* = 0.5) [18]. However, this finding was not observed in a similar finding among a larger study of 455 Caucasian women with breast cancer and 603 women without breast cancer [18,15]. Since UGTs are highly polymorphic, determining whether the UGT1A1 promoter polymorphism may have an impact on clinicopathological factors, is crucial for determining breast cancer risk, treatment response, and clinical outcome of the patient. Collectively, our research findings suggest that UGT1A1 may be an important component for determining breast cancer risk as it relates to stage, menopausal status and ethnicity; however, additional studies are warranted to confirm these findings.

## Figures and Tables

**Table 1:** Selected characteristics of donors analyzed in this study.

Characteristic	Cases (n = 57)^a^	Controls (n = 35)^a^
Age, years (mean ± SD)	49.6 ± 14.7	34.2 ± 12.6
Ethnicity, n (%)		
African American (blacks)	7 (12.3)	17 (48.6)
European American	50 (87.7)	18 (51.4)
Stage of Breast Disease		
I	9 (15.8)	
II	26 (45.6)	
III	19 (33.3)	
IV	3 (5.26)	
Menopause Status, n (%)		
Pre-menopausal	22 (38.6)	28 (80)
Post-menopausal	35 (61.4)	7 (20)

a Number of samples in each group/Total number of samples

**Table 2:** Genotype frequencies of the UGT1A1 polymorphism in breast cancer cases and controls.

*UGT1A1* (TA) genotype	Cases^a^ n (%)	Controls^a^ n (%)
UGT1A1*1/*1	33 (58)	20 (57)
UGT1A1*1/*28	6 (11)	5 (14)
UGT1A1*28/*28	13 (23)	6 (17)
UGT1A1*28/*37	5 (9)	4 (11)
Total	57	35

a Number of samples with genotype/total samples

**Table 3:** Mean mRNA expression of UGT1A1 for each genotype in breast cancer cases and controls.

UGT1A1(TA) genotype	Cases	Controls	*P*-value
UGT1A1*1/*1	0.7 ± 0.3	4.7 ± 1.6	0.004
UGT1A1*1/*28	0.2 ± 0.1	1.5 ± 1.2	0.24
UGT1A1*28/*28	0.2 ± 0.2	1.6 ± 0.8	0.03
UGT1A1*28/*37	0.01 ± 0.02	0.5 ± 0.2	0.06

Data are presented as the mean average of samples in each group ± standard deviation

*p*-value represents cases vs. controls per genotype in each row

* *p* < 0.05 is considered significant

## Abbreviations:

UGT: UDP-Glucuronosyltransferases
ER: Estrogen Receptor
TA: TATA box
